# Treatment of Idiopathic Pulmonary Fibrosis

**DOI:** 10.7759/cureus.15360

**Published:** 2021-05-31

**Authors:** Sherif T Abuserewa, Richard Duff, Gregory Becker

**Affiliations:** 1 Internal Medicine, Grand Strand Regional Medical Center, Myrtle Beach, USA; 2 Department of Pulmonary and Critical Care Medicine, Grand Strand Medical Center, Myrtle Beach, USA

**Keywords:** ipf, future management, progressive interstitial lung disease, pulmonary disease, literature review of disease

## Abstract

Idiopathic pulmonary fibrosis (IPF) is a chronic, progressive, fibrosing interstitial pneumonia of unknown cause, occurring in adults and limited to the lungs. In the past, treatment was aimed at minimizing inflammation and slowing the progression of inflammation to fibrosis. However, the underlying lesion in IPF may be more fibrotic than inflammatory, explaining why few patients respond to anti-inflammatory therapies and the prognosis remains poor.

In this review of literature, we will be focusing on main lines of treatment including current medications, supportive care, lung transplantation evaluation, and potential future strategies of treatment.

## Introduction and background

Idiopathic pulmonary fibrosis (IPF) treatment plan depends on the disease prognosis, severity, and the patient’s wishes. IPF treatment can be challenging as the disease course is unpredictable where some patients develop episodes of exacerbations after a period of stability, and the actual range of survival of these patients is highly variable where 20%-25% of patients’ survival may exceed 10 years [1-4]. Moreover, patients with IPF are usually older in age and have comorbidities such as chronic obstructive pulmonary disease (COPD) and heart failure, which may worsen their IPF symptoms. Treating physicians can have difficulty in figuring out if patients' functional limitations are the result of disease progression, comorbidities, deconditioning, or simply the aging process.

## Review

Disease severity and prognosis

The gender-age-physiology (GAP) model is the most commonly used and validated clinical prediction model that can predict 1, 2, and 3 years of mortality risk [5], and thus it is a good estimate of patient's prognosis that can be included with the patient's disease severity in the physician-patient discussion about available treatment options either medically or surgically such as lung transplantation or palliative care. Furthermore, the prognosis is reassessed along the disease course clinically by the occurrence of exacerbations or worsening of symptoms and by pulmonary function test assessment every three to six months. If there is a significant decline in pulmonary function, the available treatment options may change [6,7]. Recently, some blood and genomic markers can find their way as prognostic factors in IPF patients. Blood markers include proteins reflecting alveolar epithelial cell injury or activation of fibrotic pathways [8] including periostin, fibulin-1, collagen degradation products, CA 19-9, CA 125, and increasing circulating fibroblasts. On the other hand, genomic markers include peripheral blood gene expression profiles [9] and peripheral blood leukocyte telomere length [10].

Disease severity in IPF is assessed as illustrated in Table 1.

**Table 1:** Symptoms and signs, HRCT findings, PFTs, 6MWT, and P(A-a)O2 gradient differences in IPF patients with different disease severities PFTs, Pulmonary function tests; HRCT, high-resolution computed tomography; 6MWT, six-minute walk test; P(A-a)O_2_, pulmonary alveolar to arterial oxygen gradient; IPF, idiopathic pulmonary fibrosis.

	Mild disease	Moderate disease	Severe disease
Symptoms and Signs	Asymptomatic or mild, nonproductive cough, and dyspnea with substantial exertion	Dyspnea on moderate exertion, nonproductive cough	Dyspnea on mild exertion (e.g., walking <300 feet or climbing more than one flight of stairs)
Radiographic findings by high-resolution computed tomography (HRCT) taken at three levels (e.g., tracheal carina, inferior pulmonary veins, and 1 cm above the dome of the diaphragm) [1]	Reticular opacities and areas of honeycombing are limited to subpleural and basilar areas, involving less than 10% of the lung parenchyma	Reticular opacities involving 20%-30% of the lung and honeycombing involving <5% of the parenchyma [11]	Extensive honeycombing is noted (>5% of the parenchyma in three or more zones) [11]
Pulmonary function tests (PFTs)	Normal or may show mild reductions in forced vital capacity (FVC), diffusing capacity (DLCO)	Reduced FVC (50%-70% of predicted), a reduced DLCO (45%-65% of predicted)	Moderate to severe reductions in the FVC (<50% of predicted), DLCO (<50% of predicted)
Six-minute walk test (6MWT)	Normal or mildly reduced	Reduced and supplemental oxygen may be needed with exertion	Desaturation (≥4%) during the test [12]
Pulmonary alveolar to arterial oxygen gradient [P(A-a)O_2_]	Normal or mildly elevated (<20 mmHg)	Elevated (21-30 mmHg)	elevated (>30 mmHg)

Medical therapies

Until now, there is no curative treatment for IPF; however, nintedanib and pirfenidone are two medications working on slowing disease progression [2,4,13] and do have a mortality benefit [14]. According to the current data, neither medication is superior in terms of efficacy; and thus, choosing between either of them will depend on the patient’s tolerance to side effects. Table 2 demonstrates a detailed comparison between both medications.

**Table 2:** Comparison between nintedanib and pirfenidone

	Nintedanib	Pirfenidone
Mechanism of action	Tyrosine kinase receptor blocker preventing the release of fibrogenic growth factors and thus slows down fibrosis [15,16]. It interacts with CYP3A4 inducers and inhibitors.	Blocks transforming growth factor-beta (TGF-β)-stimulated collagen synthesis and decreases the extracellular matrix [17,18]. Inhibits proliferation of fibroblasts.
Dose and administration	150 mg every 12 hrs	40 mg/kg per day and not exceeding 2403 mg per day in three divided doses. 1 capsule is 267 mg. Starting dose is 1 capsule TID for a week; then titrate it up to be 2 capsules TID for another week, and then titrate it up to full dose 3 capsules TID.
Adverse effects	Gastrointestinal (GIT) upset including nausea, vomiting, and diarrhea [19]. Hepatotoxicity and teratogenicity [19]. Increases bleeding tendency in patients on anticoagulants.	GIT upset including anorexia, dyspepsia, nausea, abdominal pain, and diarrhea [20]. Hepatotoxicity [20], Dry skin, itching, and hyperpigmentation.
Precautions to be taken	Diarrhea can be treated with good hydration and antidiarrheal medications; if not responding, we may decrease the dose to 100 mg, and if it is still intolerable, medication should be stopped. Liver function tests should be checked before starting treatment, then monthly for 3 months and then every 3 months. Nintedanib is contraindicated in patients with moderate to severe liver disease [19]. A pregnancy test should be performed before starting the treatment, and pregnancy should be avoided throughout the treatment duration and at least for 3 months after the last dose [19].	Should be taken after meals to reduce GIT upset symptoms [21]. Liver function tests should be checked before starting treatment, then monthly for 6 months, and then every 3 months [20]. Pirfenidone is metabolized by CYP1A2, so any CYP1A2 inhibitors use should be associated with a reduction in the pirfenidone dose [20].
Efficacy	Decreased the pulmonary function decline and prolonged the time for first exacerbation per clinical trials mentioned below	Decreases pulmonary function decline and has a mortality benefit in patients with mild-to-moderate disease according to the clinical trials mentioned below.
Role in severe disease	No clinical trials were done on patients with severe disease, and only results of observational trials were reported. Reduction in pulmonary function decline was noted. However, improvement in survival or symptoms was not evident [22,23]. Side effects were more evident with higher rates of treatment interruption [22,24].	Very limited clinical trials were done on patients with severe disease. RECAP study showed the same rate of decline in pulmonary function tests in both severe and non-severe disease patients. However, clinical worsening of IPF and treatment interruption were more common in severe disease than non-severe [25,26].
Clinical trials	TOMORROW trial [27,28], INPULSIS-1, and INPULSIS-2 trials [16,29-31]	ASCEND trial [32], CAPACITY 004, and 006 trials [33]

Supportive care

Supportive care is crucial for IPF patients and considered one of the main lines of treatment in IPF. It can be categorized in the following main points:

Supplemental Oxygen

Long-term oxygen therapy (LTOT) is considered an inevitable treatment in patients with IPF with disease progression, either intermittently during exertion or sleep or continuously with severe disease. Indications for continuous LTOT depend on arterial oxygen tension (PaO_2_) and pulse oxygen saturation (SpO_2_) [34]. In general, PaO_2_ < 56 mmHg or SpO_2_ < 89 mmHg is an indication for continuous LTOT. However, in cases where there is presence of cor pulmonale, polycythemia, or right-sided heart failure, PaO_2_ < 60 mmHg or SpO_2_ < 90 mmHg is an indication for continuous LTOT. The LTOT may be indicated during sleep if the patient meets the above criteria or if PaO_2_ drops > 10 mmHg and/or SpO_2_ drops >5% with clinical evidence of nocturnal hypoxemia including insomnia, morning headaches, and impaired cognitive function. LTOT may be indicated during exercise if the patient meets the general indication of LTOT. Some studies showed that hyperoxia improves exercise tolerance in a dose-dependent manner, up to an inspired oxygen fraction of 50% [35], and thus, oxygen therapy may be indicated if the patient is having significant dyspnea during exercise even if the patient is not desaturating [36]. It is important to note, however, that oxygen is neither beneficial nor does it provide symptomatic relief as a palliative treatment in dyspnea without hypoxemia in patients with advanced lung diseases.

LTOT may improve quality of life, mental function, and exercise capacity. It can also reduce the frequency of hospitalization, cardiovascular complications, and mortality [37-39]. Most of the trials done to assess LTOT benefits were on COPD patients. More studies are needed to be applied to IPF patients.

While oxygen therapy is needed to correct hypoxemia, the oxygen flow rate should be accurately adjusted depending on PaO_2_, SpO_2_, and PaCO_2_ to maintain adequate acid-base status, which can be perfectly assessed through arterial blood gas measurements. Studies do not show an optimum level for PaO_2_ or SpO_2_, which can improve survival or quality of life. However, a PaO_2_ of 60-65 mmHg or SpO_2_ of 90%-92% is generally considered an adequate range as hypoxemia is corrected without increasing the risk of CO_2_ retention [40]. Moreover, this range has the least risk for oxidative stress caused by the oxygen therapy, which in turn causes cell remodeling, cell death, and tissue damage [41].

Pulmonary Rehabilitation

Dyspnea on exertion and poor exercise tolerance are the main symptoms of IPF. Several studies have shown that pulmonary rehabilitation can ameliorate these symptoms leading to improvement in quality of life [42-45].

Vaccination

Patients should be educated about the importance of vaccination against influenza and pneumonia as they decrease the risk and frequency of IPF exacerbations secondary to these infections. Influenza and pneumococcal polysaccharide vaccine should be offered to all IPF patients unless contraindicated.

Education and Palliative Care

Better outcomes were seen with patients who were educated about their diagnosis and management [46]. Palliative care discussion should be a part of the patient’s education as it aims to relieve suffering at all stages of the disease and is not limited to the end of life care [47]. Understanding patient's beliefs and desires is crucial for goals of care [48].

Lung transplantation

IPF is the most common interstitial lung disease among referrals for lung transplantation [49]. Understanding the type of lung transplantation, timing and indication of referral, factors putting the patient at high risk for the procedure, and physiological changes occurring post-transplantation are all crucial for determining candidates for transplantation and a proper plan of management.

After transplantation, pulmonary function tests and oxygenation improve significantly as observed in the short- and long-term follow-up of the recipients [50].

Type of Transplantation Procedure

Single lung transplantation (SLT) is considered the standard procedure for IPF [49]. After SLT, the remaining native lung tissue with low lung compliance and high vascular resistance will divert the ventilation and perfusion to the normal transplanted lung tissue. Bilateral lung transplant (BLT) is preferred if lungs with IPF have developed cysts, bullae, and bronchiectasis, which can act as a nidus for infections in the transplanted lung in SLT. BLT has shown better long-term survival and higher forced expiratory volume in one second (FEV1) in some studies when compared to SLT [51,52]. Living donor lobar lung transplantation (LDLLT) can be considered as a lifesaving option for highly critical IPF patients who may die waiting for SLT. A study was done on nine patients having LDLLT, and only one patient passed away after two lobes transplants, whereas the other eight patients’ survival was between 10 and 48 months after transplant [53].

Timing of Referral for Transplantation

IPF patients are usually the most critical patients on the transplant list with the highest death rate, that is why early referral for transplant evaluation should be done even before assessing response to initial medical treatment [54]. IPF patients should be referred for transplantation if they meet the following criteria [55] including dyspnea or functional disability related to lung disease and desaturating below 89% at rest or exertion. Moreover, diffusing capacity (DLCO) < 40% of predicted, forced vital capacity (FVC) < 80% of predicted, and decline in FVC ≥ 5%-10% and DLCO ≥ 15% during six months of follow-up are the recommended spirometry criteria. Radiologically and histologically, a picture of usual interstitial pneumonia (UIP) is needed for referral.

Placing on Transplant List and Priority on the List

Patients are usually listed on the transplant list if they meet the following: [55] hospitalization due to respiratory decline or acute exacerbation, desaturating to <88% or distance walked <250 meters or >50-meter decline in distance walked over six months. The decline in FVC ≥ 5%-10% and DLCO ≥ 15% during six months of follow-up are the required spirometry criteria. Furthermore, right heart catheterization or echocardiogram showing evidence of pulmonary hypertension is needed for the patient to be listed.

Priority on the list is determined using a lung allocation score that can assess the medical urgency and expected outcome on a scale from 1 to 100, where a higher score indicates higher urgency and better outcome with transplantation.

Factors Increasing the Risk of Transplantation

There are some factors that can put IPF patients at a higher risk of complications if they undergo lung transplants, which include pulmonary hypertension, telomerase complex mutations, and glucocorticoid therapy. Mild-to-moderate secondary pulmonary hypertension preoperatively has shown an increased risk of reperfusion injury. However, it did not show any worsening survival in two retrospective studies [52,56]. Telomerase complex mutations in IPF patients are usually associated with hematological complications including thrombocytopenia, anemia, neutropenia or severe bone marrow failure, and/or myelodysplasia with a high death rate [57,58]. Glucocorticoid therapy effect on lung transplantation differs according to the dose. Lower doses have not shown adverse outcomes, whereas higher doses may have lower survival after transplantation [59,60].

Future treatment

Currently, there is no available curative treatment for IPF. All available treatments work for a subset of patients and have significant side effects, and survival is generally poor. There are some medications under trial, which seem to be less toxic and have promising outcomes.

Combination Nintedanib Plus Pirfenidone

As discussed above, each of these medications works on slowing down IPF progression. However, the efficacy of combining both medications is still under trial. Multiple trials were done by combining both medications together with different doses, and they were found to be safe and well-tolerated by the majority of patients without major adverse effects other than expected from either medication alone [61-63].

Pentraxin 2

Pentraxin 2 is a serum amyloid believed to have an antifibrotic and anti-inflammatory effect by inhibiting differentiation of monocytes into macrophages and fibrocytes, thus halting their profibrotic and proinflammatory effects. It also inhibits transforming growth factor (TGF)-beta1 production responsible for connective tissue formation. Pentraxin 2 serum level is found to be lower in IPF patients than in normal individuals [64]. The preliminary results for a randomized trial of IPF patients receiving recombinant human pentraxin 2 intravenous (IV) in comparison with placebo showed a slower decline in pulmonary function on the pentraxin 2 side [65].

GLPG1690

GLPG1690 is a potent and selective autotaxin inhibitor, which has a good safety and tolerability profile as it has the same adverse effects when compared to placebo in a small randomized clinical trial. However, larger randomized clinical trials are needed to determine its efficacy. Autotaxin is an enzyme responsible for the conversion of lysophospholipids (lysophosphatidic acid [LPA] from the hydrolysis of lysophosphatidic choline [LPC], the latter of which is produced in the liver). LPA is believed to stimulate dendritic cell differentiation and smooth muscle contraction and prevents apoptosis. Autotaxin may in part be responsible for the “chronic wound response." Little is known about the expression and function of autotaxin, LPA, and LPC in human diseases such as pulmonary fibrosis [66].

Pamrevlumab (FG-3019)

Pamrevlumab is a monoclonal antibody targeting connective tissue growth factor, which has a role in fibrosis pathogenesis. Studies have shown that it has a good safety profile, and it slows the decline in FVC compared with pirfenidone and nintedanib. It may become a potential IPF treatment [67]. Further larger trials are needed.

Treatment for Gastroesophageal Reflux Disease

Gastroesophageal reflux disease (GERD) is a common cause of chronic cough in the general population, but it is also found to be more common in IPF patients [68,69]. Moreover, in the presence of possibility of micro-aspiration with GERD, some studies hypothesized GERD as an important risk factor for the development or worsening of IPF [70-72] and the associated cough [73]. According to this hypothesis, several studies were done to monitor the effect of anti-GERD medications and surgery on progression and outcomes of IPF.

Upon using anti-GERD medications, some studies have shown decreased radiographic fibrosis scores on chest CT scans and better survival rates [74], while others showed a smaller decline in pulmonary function tests [75]. Some observational studies have shown a reduction in IPF-related mortality but not overall mortality [76].

Studies done on IPF patients after anti-reflux surgery showed it to be a well-tolerable procedure. Larger randomized trials are needed for the efficacy of the procedure on IPF progression [77].

Medications that should not be used anymore

The following are medications that were used in the past, but studies have recommended against using them either because of no benefit or severe side effects including higher mortality.

Anticoagulation

Anticoagulation with warfarin was previously used in IPF patients due to the assumption of the existence of prothrombotic state [78] and was found to improve mortality after three years follow-up when used with prednisone in comparison to prednisone alone in a non-blinded trial [79]. However, the Anticoagulant Effectiveness in Idiopathic Pulmonary Fibrosis trial (ACE-IPF) results showed increased mortality in patients on anticoagulation for IPF without other indications for anticoagulation, and thus, anticoagulation should not be used in IPF unless there is another indication for it [4].

N-Acetylcysteine in Combination With Either Azathioprine-Prednisone or With Pirfenidone

Azathioprine-prednisone in combination with N-acetylcysteine or even N-acetylcysteine as a monotherapy has been used for many years as it was supposed to have some benefit according to the preliminary results of IFIGENIA (Idiopathic Pulmonary Fibrosis International Group Exploring (N) acetylcysteine I Annual) trial [80]. However, PANTHER trial results showed more frequent hospitalizations and adverse effects including higher mortality with this combination therapy in comparison to placebo. We recommend against using this combination treatment [81]. Moreover, a recent study has shown that combination treatment with inhaled N-acetylcysteine and pirfenidone is likely to result in worse outcomes for IPF [82].

Endothelin Receptor Antagonists

Several trials were done on endothelin receptor antagonists to check their efficacy in IPF treatment, but they did not show any significant benefit, and sometimes they were even harmful to the patient. Bosentan was used in IPF for its anti-fibrotic properties, but it did not show any improvement in exercise tolerance or quality of life according to BUILD 1 trial (Efficacy and Safety of Oral Bosentan in Patients With Idiopathic Pulmonary Fibrosis) [83] or any improvement in mortality or frequency of exacerbations according to BUILD 3 trial (Bosentan Use in Interstitial Lung Disease 3) [84]. Macitentan was also used in IPF treatment in MUSIC trial (Macitentan Use in an IPF Clinical Study), which also did not show any significant benefits [85]. Ambrisentan was tested in ARTEMIS-IPF trial for the treatment of IPF, which did not show any benefit but instead showed more frequent hospitalizations and exacerbations in comparison to placebo [86].

Phosphodiesterase Inhibitors

Trials of sildenafil in patients with IPF for improving exercise tolerance and quality of life have shown minimal benefit [87,88], so sildenafil is not advised for routine use in IPF.

Methotrexate

Methotrexate use did not show any benefit in the treatment of IPF, but instead, sometimes it induced pneumonitis that cannot be differentiated from an IPF exacerbation. However, methotrexate may be of benefit if IPF resulted from an autoimmune disease that can be treated with methotrexate, for example, as in rheumatoid arthritis [89].

Colchicine [90-92], cyclophosphamide [93], cyclosporine [94], etanercept [95], interferon gamma-1b [96], penicillamine [92], and simtuzumab [97] were studied separately in multiple studies, but none of them showed significant benefit or improvement in outcome. Therefore none of the aforementioned medications are recommended for the treatment of IPF.

## Conclusions

This is a review of literature on the treatment options of stable IPF. We discussed the different severities and prognosis of IPF according to the clinical picture, radiographic findings, PFTs, 6 MWT, and P(A-a)O2. Nintedanib and pirfenidone are two medications that work on slowing disease progression and having a mortality benefit. However, supportive care is crucial for IPF patients and considered one of the main lines of treatment in IPF including supplemental oxygen, pulmonary rehabilitation, vaccination, patients' education, and palliative care discussions. IPF is the most common interstitial lung disease among referrals for lung transplantation. These patients are usually the most critical patients on the transplant list with the highest death rate. However, they need to fulfill certain criteria for lung transplantation referral and listing on priority list. Currently, there is no available curative treatment for IPF. There are some medications under trial, which seem to be less toxic and have promising outcomes including combination nintedanib plus pirfenidone, treatment for GERD, Pentraxin 2, and others. Some other medications were used before, but studies have recommended against them either due to no benefit or severe side effects including higher mortality. These therapies include anticoagulation, azathioprine-prednisone-N-acetylcysteine, endothelin receptor antagonists, phosphodiesterase inhibitors, methotrexate, and others.
